# Neonicotinoids Interfere with Specific Components of Navigation in Honeybees

**DOI:** 10.1371/journal.pone.0091364

**Published:** 2014-03-19

**Authors:** Johannes Fischer, Teresa Müller, Anne-Kathrin Spatz, Uwe Greggers, Bernd Grünewald, Randolf Menzel

**Affiliations:** 1 Institut für Bienenkunde Oberursel, Polytechnische Gesellschaft Frankfurt am Main, Fachbereich Biowissenschaften, Goethe-Universität, Frankfurt am Main, Germany; 2 Netzwerk Blühende Landschaften Fischermühle, Rosenfeld, Germany; 3 Institut für Biologie, Freie Universität Berlin, Berlin, Germany; Colorado State University, United States of America

## Abstract

Three neonicotinoids, imidacloprid, clothianidin and thiacloprid, agonists of the nicotinic acetylcholine receptor in the central brain of insects, were applied at non-lethal doses in order to test their effects on honeybee navigation. A catch-and-release experimental design was applied in which feeder trained bees were caught when arriving at the feeder, treated with one of the neonicotinoids, and released 1.5 hours later at a remote site. The flight paths of individual bees were tracked with harmonic radar. The initial flight phase controlled by the recently acquired navigation memory (vector memory) was less compromised than the second phase that leads the animal back to the hive (homing flight). The rate of successful return was significantly lower in treated bees, the probability of a correct turn at a salient landscape structure was reduced, and less directed flights during homing flights were performed. Since the homing phase in catch-and-release experiments documents the ability of a foraging honeybee to activate a remote memory acquired during its exploratory orientation flights, we conclude that non-lethal doses of the three neonicotinoids tested either block the retrieval of exploratory navigation memory or alter this form of navigation memory. These findings are discussed in the context of the application of neonicotinoids in plant protection.

## Introduction

Bees navigate in a range of several kilometers around their hive and communicate about locations using the waggle dance to transmit information about the flight vector towards a feeding place or a nest site [1], [2]. Navigation and communication require multiple cognitive faculties. Among these are, for example, recognition of the sun compass, visual distance estimation, learning of multisensory cues inside and outside the hive and translating as well as reading the codes of the waggle dance, all processes that require integration of different navigational information. Several forms of memory have been found to guide navigation (recent reviews: [3], [4], [5], [6]). In particular, route flights between hive and feeder lead to a memory of the flight vector that are bound to the sun compass and can be communicated in the waggle dance. A more flexible memory about spatial relations of landmarks is formed during the exploratory orientations flights of bees leaving the hive for the first time. This latter form of memory allows bees to steer to learned locations e.g. the hive, a different feeding site, and a dance communicated site along novel short cutting routes. It has been concluded that that these multiple spatial representations are integrated in a common frame of spatial reference. Insecticides acting on neural functions of the insect brain are possibly compromising the sensory, motor and central processing required for these cognitive functions [7], [8], [9], [10], [11], [12]. Here we ask whether non-lethal doses of three neonicotinoids (imidacloprid, clothianidin, thiacloprid) interfere with navigational performance during returning flights after relocation during a foraging trip.

Neonicotinoids are insecticides widely used in agriculture to protect crops against pest species. They act as agonists of the insect neuronal nicotinic acetylcholine receptors (nAChR). Since they exhibit much lower affinity to the mammalian nAChRs [13], they are rather specific (review [14]). Unlike acetylcholine the neonicotinoids are not degraded by the enzyme acetyl cholinesterase. Binding of the neonicotinoid at the nAChRs will activate the receptor and via depolarization cause excitation of the postsynaptic membrane (e.g. [15]–[17], [18], [19], [20]). Thus, signal transmission via the neuronal insect nAChR is disturbed either by continuous synaptic stimulation or by blocking the binding of the natural transmitter, acetylcholine. The excitation of nAChRs even at sublethal doses leads to muscle cramps, activation and paralysis, and is suspected to interfere with central nervous processing. Acetylcholine is the most abundant excitatory transmitter in the insect central nervous system. Within the brain cholinergic synaptic transmission is suggested to occur from axons of the olfactory receptor neurons onto local interneurons and projection neurons within the antennal lobes (in addition some of the local interneurons are cholinergic), from antennal lobe projection neurons onto mushroom body Kenyon cells and onto neurons of the lateral horn, between ocellar second-order neurons and their postsynaptic neurons. In addition, the insect central brain, optic lobes and the thoracic ganglia contain cholinergic neurons (e. g., *Manduca sexta*: [21], *Schistocerca gregaria*: [22], *Periplaneta americana*: [23], *Drosophila melanogaster*: [24], [25], [26], [27], *Apis mellifera*: [28]). Given this wide-spread central nervous distribution, it is not surprising that sublethal neonicotinoid doses compromise behavior and cognitive abilities also in honeybees including memory formation and retrieval [29], [30], social interactions, navigation and communication [11], [12] [31]. Sublethal behavioral effects on pollinating bees may thus be the most likely exposure scenario in agriculture from neonicotinoid plant treatment. Although the concentrations detected in pollen and nectar from seed-treated crops with neonicotinoids are generally too low to cause immediate death from acute poisoning [32], [33], neonicotinoid residues in pollen and nectar often lead to long-term pesticide exposure when honeybees are foraging on treated crops.

Our study aims to elucidate acute effects of three neonicotinoids at doses that cause no obvious modification of bees sensory or motor performance during a natural test condition of foraging. The doses applied are somewhat higher than those expected under agricultural conditions [32], [33]. Honeybees trained to an artificial feeding site after performing their exploratory orientation flights navigate back to their hive after being caught at the feeding site on departure, transported to a release site within their explored area and released there (catch-and-release experiment [34]). In a catch-and-release situation bees fly first along a vector they would have taken if not removed from the feeding site. Then they perform search flights which end in a rather straight flight towards the hive. The first flight component will be called vector flight and the second homing flight. These two flight components refer to different navigational memories. The vector flight is based on the current memory store which the animal applies during its multiple route flights between the hive and a feeding place. The homing flight requires activation of a remote memory acquired during orientation flights and possibly other flight performances, and involves some form of localization relative to the hive [5]. Here we find that the tested neonicotinoids affect these two navigation memories differently. Vector flights showed only slight alterations but homing flights are compromised depending on the particular neonicotinoid and its administered dose. These results indicate selective actions of neonicotinoids on higher level processing in the honeybee brain.

## Materials and Methods

### Training procedure

**Figure 1:**
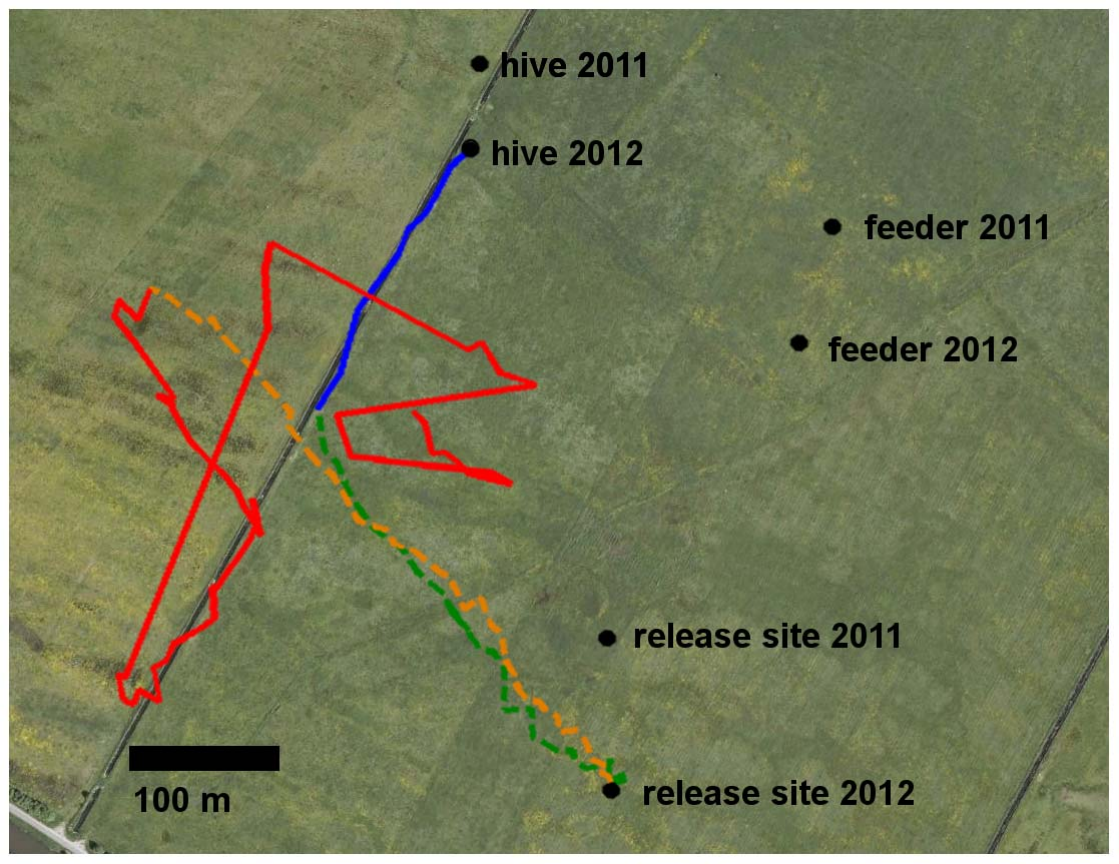
Examples of flight paths of two individual bees. The dashed lines (green, yellow) depict the vector flight component and the blue and red lines the homing flight component. The green and blue traces come from a control bee, the yellow and red traces from a bee treated with thiacloprid. The initial flight path from the release site towards west-northwest (dashed lines) is called the vector flight because it predominantly resembles the flight vector the animals would have taken if they had not been transported to the release site (catch-and-release design of experiment). The following flight path (full lines) is called the homing phase since in most animals the flight ended at the hive. If an animal did not reach the hive (as in this case after thiacloprid treatment) the homing phase was analyzed until the animals were not seen any more at the radar screen. Both bees were released at the same release site (release site 2012) and both bees showed a similar flight vector at first. Notice that the locations of the hives, the feeding sites and the release sites differed somewhat in the two experimental seasons (2010, 2011). The map was created using Google Earth (Google Inc. 2012). A scale bar is shown for 100 meter.

A group of 15–20 bees (*Apis mellifera carnica*) from a full colony (>30,000 bees) were trained to a feeding site 250 m east of the hive in an open field 1 km west of the town of Wittenberge (Brandenburg, Germany, coordinates: N 52.97555, E 11.83677). No permission was required to work in this area. The grassland is privately owned by Frau and Herr Nickel, An der Kirche 8, 19322 Klein Lüben (Brandenburg, Germany).The use of the privately owned land was permitted. No endangered or protected species were involved. No protected area was used. All trained bees were marked with colored number tags on the dorsal thorax, and a full protocol of the visits to the feeder was recorded for all bees. The test bees where caught at the feeder before they were able to drink and quickly transferred individually to a small container equipped with a miniature feeder providing 49 µl of a sucrose solution plus 1 µl of the neonicotinoids clothianidin, imidacloprid or thiacloprid. The bees were kept in a dark Styrofoam box for 90 minutes (incubation) during which they imbibed all of the sucrose solution. Six bees were caught at 15 minutes intervals in the morning for the experiments of the same day. In the afternoon, these bees were transported to the release site 450 m south of the feeding site and individually fed with the respective solution. After incubation they were put into a special holding device with a mesh at the top to attach a radar transponder onto the thorax. The transponder was glued to the number tag with a double sticky tape. Immediately afterwards the bees were released at intervals of 15 minutes to ensure the same incubation time in each bee. Care was taken that the control group and the treatment groups were evenly distributed each day. In the afternoon, during the recordings, three people worked together, one released the bees, another one ran the radar device and a third person waited at the hive for the bee to arrive. This person caught the bee, removed the transponder and killed it. Thus, it was ensured that each bee was used only once in the experiment. The person at the radar station informed the person at the hive when the bee had almost reached the hive. Data were collected during two experimental seasons (2011, 2012). Since we did not observe any differences in the flight behaviors between the years, we pooled the data were appropriate. The location of the hive and the feeder were slightly different (Fig. 1). The total number of bees tested was 98 in 2011 and 110 in 2012. Representative examples of the flights tracks are shown in the Supporting Information with the file names referring to the five experimental groups (Archive S1, Archive S2, Archive S3, Archive S4 and Archive S5).

The following measures were taken: release time, start time of flying, arrival time at the hive and the flight trace recorded with the harmonic radar. From these measures the following parameters were derived for each bee: departing/not departing bee (if a bee did not depart, was observed sitting in the grass for longer than 30 minutes, or was never seen on the radar then it was classified as non-departing), immediate/delayed departure (if a bee delayed its departure by up to 15 minutes and was then seen on the radar then it was classified as a delayed departure), arriving/non-arriving bee (if a bee was observed by radar but disappeared from the radar and was not seen arriving on the same day then it was classified as non- arriving). In addition, the readings from the radar trace consisted in flight time, flight length, flight speed, directedness of the initial vector flight component and of the homing component. The transition from the vector flight to the homing flight was characterized by an angular turn >60° allowing to define the end of the vector flight and the beginning of the homing flight.

### Substances

Three drugs were tested during the experiment: clothianidin, imidacloprid and thiacloprid (thiacloprid: Bayer Crop Science Deutschland, Monheim, clothianidin and imidacloprid: Sigma Aldrich, Hamburg Germany). All three neonicotinoids were first dissolved in acetone (final concentration in the feeding solution was 0.005%–0.01%). The clothianidin and imidacloprid solutions were further diluted in water. Finally, all solutions were diluted 1 to 9 with 2 M sugar water. The final concentrations were: clothianidin (0.2 µM), imidacloprid (0.6 µM and 0.9 µM), thiacloprid (0.1 mM) leading to doses of 2.5 ng/bee (equivalent to 25 ppb) of clothianidin, 7.5 ng/bee (equivalent to 75 ppb), and 11.25 ng/bee (equivalent to 112.5 ppb) of imidacloprid and 1.25 µg/bee (equivalent to 12.5 ppm) of thiacloprid. The considerable higher thiacloprid dose was chosen due to the higher resistance of bees to this particular neonicotinoid, as reflected in the higher LD_50_.Thiacloprid was not tested in 2011. Imidacloprid at 11.25 ng was only tested in 2011, the higher dose was omitted in 2012. This resulted in a lower number of tested individuals for this dose. The sucrose solution given to the control bees contained 0.01% acetone. We compared the return rates between control bees of our experiments with the control bees in a parallel running experiment that were trained from the same hive to the same feeder and released in parallel from the same release site without acetone in the sucrose solution [38] and found no difference (88% in our control bees and 90% in the control, bees of [38]).

### Harmonic radar tracking

Tracking bees with a harmonic radar system is described in [35], [36], [37]. We used a system with a sending unit consisting of 9.4 GHz radar transceiver (Raytheon Marine GmbH, Kiel, NSC 2525/7 XU) combined with a parabolic antenna providing approx. 44 dBi providing a signal from the transponder on the bee thorax every 3 s. The transponder consisted of a dipole antenna with a Low Barrier Schottky Diode HSCH-5340 of centered inductivity. The second harmonic component of the signal (18.8 GHz) was the target for the radar. The receiving unit consisted of an 18.8 GHz parabolic antenna, with a low-noise pre-amplifier directly coupled to a mixer (18.8 GHz oscillator), and a downstream amplifier with a 90 MHz ZF-Filter. A 60 MHz ZF-Signal was used for signal recognition. The transponder was made of a silver or gold wire with a diameter of .3 mm, a length of 11 mm, a weight of 10.5 mg and a loop inductance of 1.3 nH. The range of the harmonic radar was 1 km radius. Occasionally radar signals were missing as identified by a time interval between two consecutive radar signals >3 s. In such a case a surrogate signal was produced by assuming equal distance along a straight line between the two adjacent signals. Representative examples of flights tracks are given in the Supporting Information in file archives for each of the five experimental groups (Archive S1, control group; Archive S2, clothianidin treatment; Archive S3, imidacloprid 0.6 µM treatment, Archive S4, imidacloprid 0.9 µM treatment; Archive S5, thiacloprid treatment). The x- and y-axis is scaled in meters and the 0/0 coordinate marks the radar position.

### Analysis of the flight tracks and statistical analysis

We acquired recordings for each bee separately, consisting of x/y coordinates for distinct timepoints of the radar signals. These were used to reconstruct the flight path of the corresponding bee. All flights consisted of more than 15 data points per bee. Additionally, we recorded the time of departure and arrival (if the bee arrived at the hive). Non- circular statistics were done with Matlab v.R2011b (The MathWorks, Inc., USA). We used Barnard's Exact Probability Test for comparison of arriving or not arriving bees. Data for flight time and length were tested for normal distribution with the Lilliefors test. We found in each variable group at least one treatment group with non-parametric data. Therefore, we used a Kruskal-Wallis multi comparison between the groups with a Scheffe correction to find differences in the groups. This was followed by a group to group comparison using a Wilcoxon Ranksum test.

The circular statistics for comparison of the angles for the different treatments was done with Oriana v4 (Kovach Computing Services, Wales, U.K.). Angular deviation was calculated with the Watson -Williams F-test, distribution for angular data between groups was tested with the Mardia-Watson-Wheeler test.

## Results

### Global analysis

In a catch-and-release experiment as applied here bees were trained first to forage at a feeder for at least 2 days (Fig. 1), and were then caught at the feeder upon arrival, transferred to a container equipped with a miniature feeder containing a defined volume of sucrose solution with or without the neonicotinoid to be tested. When released at a remote release site 1.5 hours later bees frequently settled in the grass for a while, then performed a few narrow circular flights and flew straight to the west resembling the vector they would have taken from the feeder to the hive (vector flight, Fig. 1, see also Archive S1, Archive S2, Archive S3, Archive S4 and Archive S5). The experimental setting in our experiments brought the vector flight of the bees close to an extended landmark, a narrow irrigation channel stretching approximately south-north. Well oriented bees took a sharp turn at the end of their vector flight to the north close to this landmark and flew straight back to the hive which was located close to this landmark in the north of the test field. Less well oriented bees ended their vector flights also by a sharp turn but then started search flights before and during their flight back to the hive. Thus the flight track of a bee from the release site to the hive can be divided into two phases, the vector flight and the homing flight.

If bees did not start immediately they settled in the grass and could be observed until they departed. Since we preselected the dose of the tested neonicotinoids such that the treated bees were able to fly after 90 min incubation only a small number of bees did not start to fly, and there were no significant differences between the control and the treatment groups (control: 1 of 57, clothianidin: 1 of 55, imidacloprid 0.6 µM: 2 of 58, imidacloprid 0.9 µM: 2 of 19, thiacloprid: 3 of 27). However, significantly more delayed starts were found in thiacloprid treated bees (p<0.05, Barnard's Exact Probability test, see Table 1). The proportion of bees returning to the hive differed between the control groups, and 3 out of the 4 treated groups. Fifty out of 57 (88%) control bees successfully returned to the hive. The respective numbers of the neonicotinoid treated bees are: clothianidin (0.2 µM): 43 (n = 55, 78%); imidacloprid (0.6 µM): 42 (n = 58, 72%); imidacloprid (0.9 µm): 6 (n = 19, 32%); thiacloprid (0.1 mM): 12 (n = 27, 44%). Treatment with either imidacloprid (0.6 µM), imidacloprid (0.9 µM) or thiacloprid (0.1 mM) significantly increased the number of bees failing to return to the hive (p<0.05; Barnard's Exact Probability test) although they departed from the relase site and did not show any obvious changes in their flight behavior.

**Table 1:** Overview of the total number of bees released, the number of bees that returned to the hive, the “non starting bees” and bees that delayed their start.

treatment	total number of bees	not started	arrived at the hive	not arrived
control	57	1	50	7
clothianidin (0.2 µM)	55	1	43	12
imidacloprid (0.6 µM)	58	2	42	16
imidacloprid (0.9 µM)	19	2	6	13
thiacloprid (0.1 mM)	27	3	12	15

### Vector fligth

**Figure 2:**
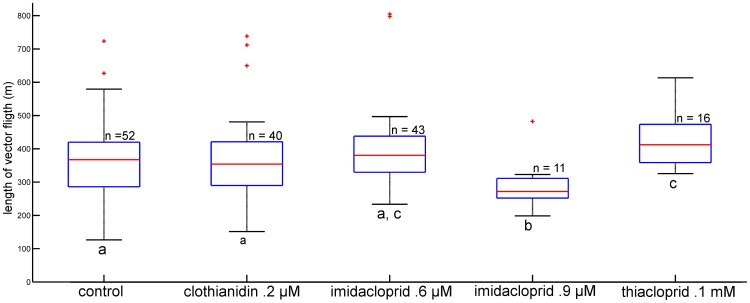
Length of vector flights for all treatment groups in meters. Groups are shown as boxplots with the median indicated in red, the edges of the box indicates the 25^th^ and 75^th^ percentiles. Outliers are shown as red crosses. Groups with no significant difference share the same letter under the lower whisker. Animals treated with imidacloprid 0.9 µM performed significant shorter vector flights than those of the control group, the clothianidin treated group, those treated with the lower concentration of imidacloprid, as well as animals treated with thiacloprid. . The thiacloprid treatment led to significantly longer vector flights compared to bees from the control group, the clothianidin group and the animals treated with the higher concentration of imidacloprid (p<0.05, Rank-sum test).

**Figure 3:**
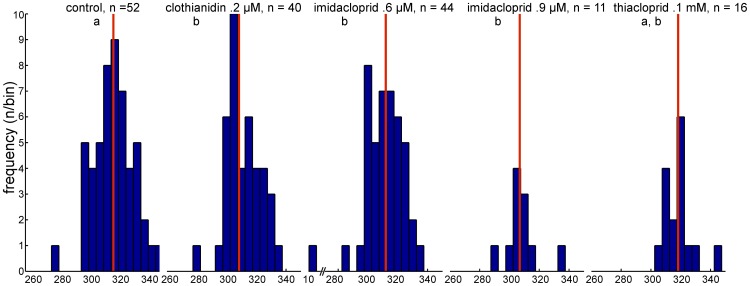
Direction of vector flights. The direction is defined by the intersection of the vector flight with a 200° bins. The x-axes give the angels in degrees clockwise from north. The median angle is marked in red for each group. Significant differences in the direction are indicated by different letters under the group names. Clothianidin treated and both imidacloprid treated groups differ significant from the control group but not from each other. The thiacloprid treated group did not differ significantly from all other group (p<0.05, Watson -Williams F-test). The direction of the learned route from the feeder to the hive is 294°, and the direct route from the release site to the hive would be 343°. Note that the x-axis is interrupted for the imidacloprid 0.6 µM group as there was one bee flying north-east with a 10.9° angle from north.

The analysis of the length of the vector flight showed a significantly shorter length for the imidacloprid 0.9 µM treated bees (p = .0242) and a longer length for the thiacloprid treated bees (p  = .0275 rank-sum test) as compared to the control group (Fig. 2.). Duration of the vector flights did not differ significantly between the control groups and the respective treatment groups (data not shown) indicating that longer vector flights were compensated for by higher flight speed. The directional components of the vector flights as indicated by their intersections with a 200 m radius around the release site are shown in Fig. 3. The angle from the feeder to the hive was 294°, from the release site to the hive 343°. The average directions of the vector flights for the control bees is 319° and for bees treated with clothianidin: 311°, with imidacloprid 0.6 µM: 313°, with imidacloprid 0.9 µM: 308°, and with thiacloprid: 317°. Thus the angular distribution for the control bees is skewed towards the shortest direction to the hive indicating that these bees may have initiated a homing component already during the vector flight. Clothianidin and both imidacloprid treatments resulted in a significant difference in the direction compared to the control group (p<0.05; Watson -Williams F-test).

**Figure 4:**
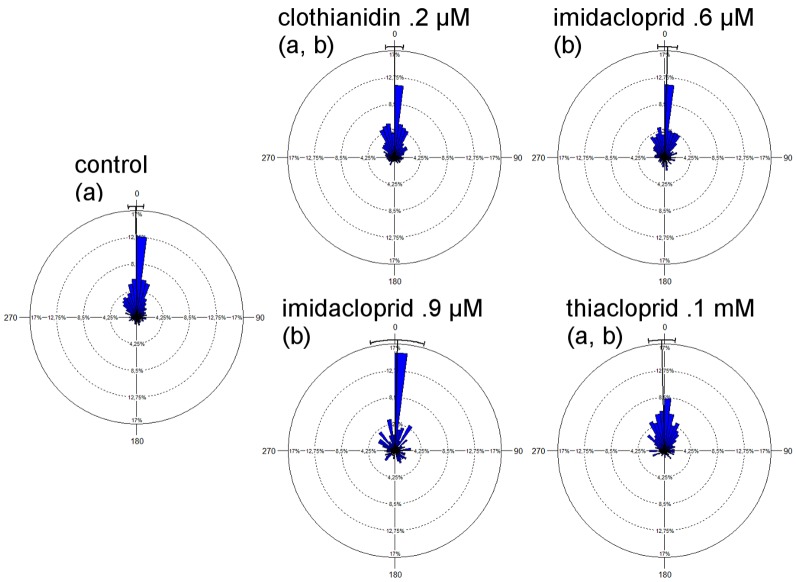
Distribution of directional changes during vector flights. Data are grouped in 50 segments ( = 7,2° each segment). Bars are scaled as percentages from 0% to 17% of the cumulative data. The black line shows the mean direction with standard deviation. Angles are given in relation to the direction of the stretch of flight shortly before, and are not related to a geographic direction (see text). The main component in all groups lies around the 0° direction indicating that the bees flew rather straight. Significant differences between the groups are shown by different letters in the parenthesis. Both imidacloprid treatments (0.6 and 0.9 µM) led to broader distributions of directions and thereby more changes in the flight path as compared to the control group. (p<0.05, Mardia-Watson-Wheeler Test).

We next analyzed the directedness of the vector flights. The directional changes during the vector flight were calculated as angles between two consecutive radar locations and expressed as deviations from the direction of the last line between two consecutive radar locations. Thus small angles indicate few changes in the flight direction, regardless of the general direction in which the bee was currently flying, and a broad data distribution indicates frequent changes in direction (Fig. 4). Both imidacloprid-treated groups showed more directional changes as compared to the control group (imidacloprid 0.6 µM p = .001; imidacloprid 0.9 µM p = .011, Mardia-Watson-Wheeler test). The broadest distribution of direction changes was found in the thiacloprid-treated group, indicating frequent directional changes. Thus, the vector flights performed by treated bees (besides those treated with clothianidin) were less straight than those of the control bees. Taken together these findings indicate that bees after neonicotinoid treatment controlled their vector flights performance less well and relied more on the sun compass related direction of their foraging flights.

### Homing flight

All animals performed a sharp turn (>60°) at the end of the vector flight. We classified the directions in which the bee initially chose to fly after this turn, and separated them into northerly, southerly (along the channel), and into other directions (Table 2). The shortest flight paths (except for a direct flight from the release site to the hive in a control bee) resulted in L-shaped flights with a sharp turn towards north. Many bees followed this flight pattern, and no significant difference was found between the control group and the bees treated with clothianidin or the two doses of imidacloprid. However, thiacloprid-treated bees had a significantly lower probability to perform L-shaped flights (p<0.05; Barnard's Exact Probability test, Table 2) and an increased probability to perform other types of flights.

**Table 2:** Flight direction after the end of the vector flight.

	flight direction after the end of vector flight				percentage of L-type flights of north flying bees
treatment	north	south	search circle	east/west	
control (n = 48)	31	14	3	0	74%
clothianidin (0.2 µM, n = 41)	29	12	0	0	62%
imidacloprid (0.6 µM, n = 41)	31	5	1	4	74%
imidacloprid (0.9 µM, n = 9)	7	0	0	2	57%
thiacloprid (0.1 mM, n = 14)	5	5	4	3	60%

The sharp turns (60°) were categorized as leading to a northerly (column north) or southerly (column south) direction, or any other direction (e.g. returning to the release site or continuing the vector flight with only a minor correction). Three thiacloprid bees (column other direction) terminated their flight at the end of the vector.

**Figure 5:**
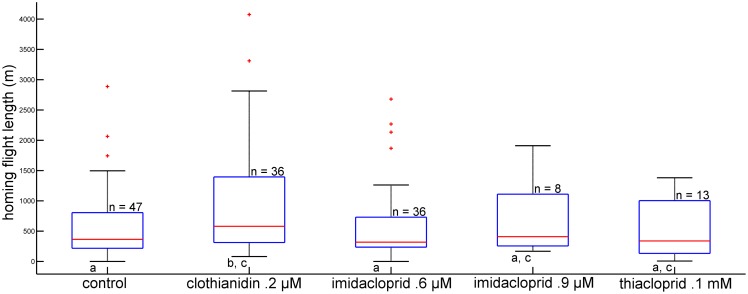
Cumulated lengths of flights during the homing phase. The homing phase started at the end of the vector flight as characterized by a turn of >60° during the vector flight and ended when the bee either arrived at the hive or was not recorded with the radar anymore. Groups are shown as boxplots with the median indicated in red, the edges of the box indicates the 25^th^ and 75^th^ percentile. Groups with no significant difference share the same letter under the lower whisker. Only clothianidin treatment resulted in a significant longer flight during the homing phase, compared to the control group and the imidacloprid 0.6 µM group (p<0.05, Rank-sum test).

**Figure 6:**
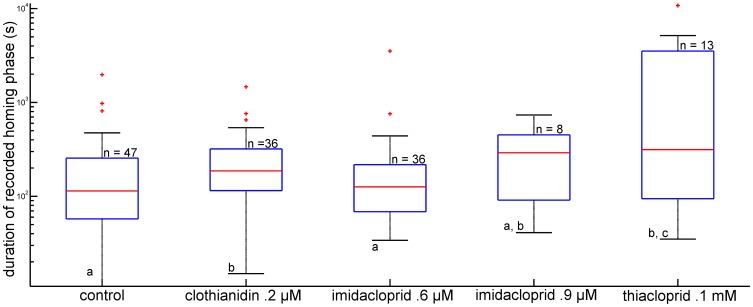
Duration of the homing phase. The homing phase started at the end of the vector flight as characterized by a turn of >60° during the vector flight and ended when the bee either arrived at the hive or was not recorded with the radar anymore. Groups are shown as boxplots with the median indicated in red, the edges of the box indicates the 25^th^ and 75^th^ percentile. Note that the y-axis is scaled logarithmically. Significant differences between the groups are shown by different letters at the bottom of each boxplot. Clothianidin 0.2 µM treatment resulted in a longer homing phase as compared to the control group and the imidacloprid 0.6 µM group. The median homing duration of the bees treated with 0.1 mM thiacloprid was significantly longer than the control group (p<0.05, Rank-sum test).

Both imidacloprid- and thiacloprid-treated bees were less successful reaching the hive during the final phase of homing. Further evaluation of the homing phase requires considering the fact that the probability of bees successfully returning to the hive differs between the control group and 3 of the 4 treatment groups (both imidacloprid- and thiacloprid-treated groups). We therefore analyzed further the flight path as recorded by the radar, without taking into account whether or not the animal arrived at the hive. Since none of the animals flew out of the range of the radar any loss of radar signals outside of the vicinity of the hive indicated landing and failure to find the hive. These animals were not captured by the person at the hive, whereas animals tracked until close to the hive were always captured by the person at the hive. The total flight path during the homing phase had a significantly longer length in bees treated with clothianidin (p<0.05, Ranksum–test) (Fig. 5), and the duration of the recorded homing flight was increased for clothianidin and thiacloprid (p<0.05, Ranksum– test) (Fig. 6). The flight speed of thiacloprid-treated bees is lower than that of all other groups (p<0.05, Ranksum– test).

The highest increase in flight duration was caused by bees that landed in the grass. In this case the recording of the radar track was stopped at the location where the individuals landed, and later resumed at this area. These interruptions in the flight where found in 6 animals from the thiacloprid treated group and lasted for more than 1500 seconds. Similar but shorter interruptions in the flight where also found in the other treatments but only for <500 seconds (5 control bees, 3 clothianidin bees, 4 imidacloprid 0.6 µM bees, none imidacloprid 0.9 µM bees). Few bees were not recorded until their arrival at the hive but returned to the feeder at the next or the over next day. This was the case for one control bee (arrived on the next day), 4 thiacloprid treated bees (1 arrived on the next day and 3 days later), 2 clothianidin treated bees (arrived next day) and 3 imidacloprid 0.6 µM treated bees (arrived next day).

**Figure 7:**
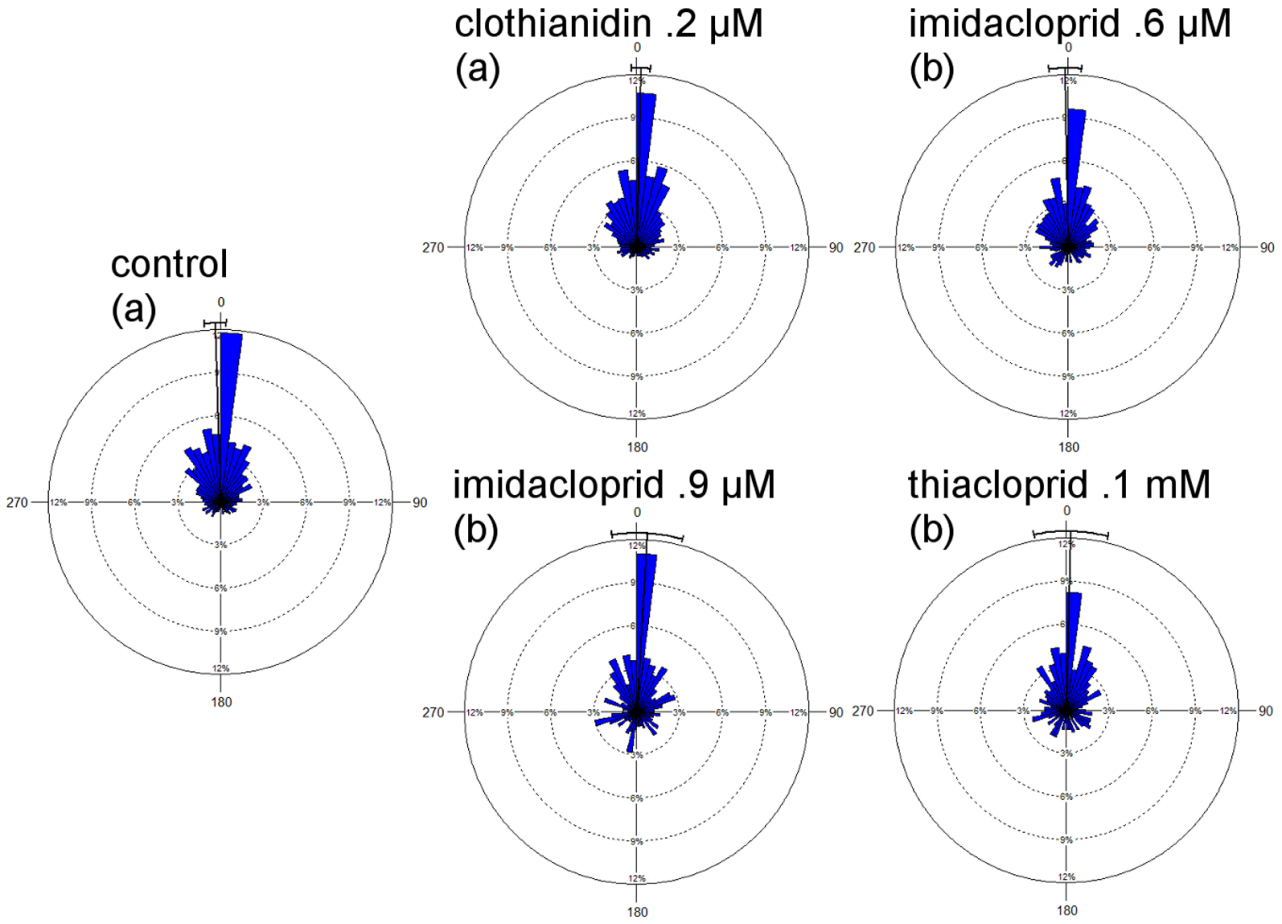
Distribution of directional changes during the homing flight. Data are shown in percent and are grouped in 50 segments ( = 7,2° each segment). Bars are scaled as percentages from 0% to 12% of the cumulative data. The black line shows the mean direction with standard deviation. Like in Fig. 4 all angles are in relation to the direction that the bee already flew, and are not related to a geographic direction. Thus the figure shows the straightness of the bees' homing flights. Significant differences were found between the control group and the group treated with clothianidin on the one side and both imidacloprid treated (0.6 and 0.9 µM) and thiacloprid 0.1 mM treated groups on the other side (p<0.05, Mardia-Watson-Wheeler test). These latter groups showed a broader spread of directions than the control and the clothianidin group, and did not differ from each other.

As expected the directedness of homing flights was lower than that of the vector flights (Fig. 7, compare with Fig. 4). A broader distribution of the directional changes in the homing phase than in the vector phase was found for the control group and the thiacloprid-treated group, and the clothianidin-treated group showed no difference. Both imidacloprid treatments led to a tendency towards broader distribution. Comparing the directedness of the homing flights between the experimental groups we found significantly lower directedness in both imidacloprid-treated groups and in the thiacloprid-treated group as compared with the control group (p<0.05, Mardia-Watson-Wheeler Test).

## Discussion

Radar tracking of honeybee flights in a catch-and-release experiment uncovers two navigational components, the initial vector flight and the ensuing homing flight. The vector component would have brought the animal back to the hive were it not transported to an unexpected release site [34].The sun compass is the dominant guiding factor in a rather even landscape as the one used here which lacked a panorama and any beacon close to the hive [34], [37], [38]. However, the ground structure of the pasture provided additional information for being south of the hive and this information was used by the control animals but not by the treated animals since the directions of the vector flights were skewed to north for the controls. Thus the treated animals were more precise in replaying the directional component of the vector flight. Furthermore, the differences in flight speed and directedness of vector flight between control and treated animals indicates more dourness for replaying the flight performance they would have applied if not transported. Thus treated bees replay their recent navigation memory more stereotypically. Flight speed during vector flights in thiacloprid-treated bees was lower than that of all other groups, indicating either an effect on flight performance or a form of reduced activation of the vector memory.

Stronger effects of neonicotinoid treatment became apparent during homing flights. The transition from vector flight to homing flight requires the activation of a different reference system, namely that which was established earlier in the life of a forager during its exploratory orientation flights and possibly during natural foraging flights. The latter is rather unlikely because no natural food sources were available in the south of the hive during the experimental period. Since the bees arrived at an unexpected location at the end of their vector flight they needed to determine in which direction the hive might be located. The experimental conditions selected for our experiments were chosen to exclude several navigation mechanisms for the bees. They could not use path integration since the animal was transported to an unexpected release site and had not the path information of this displacement. Furthermore, navigation towards a beacon at the hive and a sequential matching strategy with respect to the panorama was not possible, due to a lack of the necessary distant visual cues [34]. However, it offered a strong cue, namely the irrigation channel close to which the hive was located north of the region where the vector flights ended (see Fig. 1).Thus the test conditions made it rather easy for the bees to find the hive. Even under these conditions treated bees were significantly compromised in successful homing. The strongest effect was seen for thiacloprid-treated bees, which also showed the strongest effect on flight speed during vector flights.

The doses of neonicotinoids applied here (imidacloprid 7.5 or 11.25 ng/bee, clothianidin 2.5 ng/bee, thiacloprid 1.25 µg/bee) were selected on the finding that the treated bees were able to fly 90 minutes after starting to imbibe the solution, and to depart from the release site without obvious changes of their flight behavior. Thus, our study comprises a behavioral-toxicological approach and not an eco-toxicological approach. Nevertheless it will be interesting to compare the doses used here with those used by other authors on the basis of estimates about the doses of the respective neonicotinoids expected to be taken up by bees in an agricultural environment. Our doses of imidacloprid and clothianidin were close to the highest doses tested by Schneider et al. [8] and Henry et al. [9]. Furthermore, Whitehorn et al. [39] who fed bumble bees with pollen containing 6 µg/kg and sugar water containing 0.7 µg/l imidacloprid found significant depressing effects on several parameters of their natural development (queen production, growth rate). The authors reported that the doses were selected on the basis of findings in the agricultural conditions. Gill et al. [10] exposed bumblebees to two pesticides (neonicotinoid and pyrethroid) at concentrations that could approximate field-level exposure and detected impaired natural foraging behaviour and worker mortality leading to significant reductions in brood development and colony success. Clothianidin of 10 ppb is often exceeded in pollen carried back by foragers, and a value of 88 ppb has been measured [40]. It has been estimated that nectar collected by bees on oil rape flowers whose seeds were treated with imidacloprid contains on average (with very large variance) about 10 ppb [32], [33], which is approximately 15 times less than the lower doses of imidacloprid used in our study. Thus, 15 foraging trips of bees to such oil rape flowers combined with full absorption of the collected nectar would lead to a similar dose as in our study under the assumptions that the pesticides are fully absorbed and are not metabolized substantially. Thus, the doses in our study and those of [8], [9] can be considered to reflect a worse case as compared to those taken up by an individual bee during one foraging trip (see also EFSA Journal 201210(6) 2752). Although the debate about the relevance of the doses in behavioral-toxicological studies for the evaluation of environmental hazards through neonicotinoids is not settled [40], [41] it is obvious that the doses in these studies are not far from what one would expect for bees foraging on the flowers of treated plants.

In honeybees nicotinic acetylcholine receptors (nAChRs) targeted by neonicotinoids are involved in multiple neural nets of sensory integration and higher order information processing. Eleven candidate nAChRs subunits were identified in the honeybee genome with 9 putative alpha and 2 beta subunits (Amelα1–9, Amelβ1–2) [42]. In situ hybridization showed that 4 subunits are differentially expressed within the honeybee brain [43], [44]: the Amelα8 subunit is expressed in pupal Kenyon cells and antennal lobe neurons, Amelα5 and Amelα7 in type II Kenyon cells of the mushroom body and in antennal lobe neurons of the adult brain, Amelα2 in type I and type II Kenyon cells but not in the antennal lobes, and Amelα7 in type I KCs. Although the physiological and pharmacological properties as well as the stoichiometry of the various honeybee nAChRs is still unknown, it is obvious from behavioral experiments using nicotinergic antagonists that the honeybee nAChRs are involved in olfactory learning and memory formation [45], [46]. Furthermore, these studies indicated that 2 pharmacologically different nAChRs are differentially involved during olfactory learning and memory [45], [47]. The honeybee nAChRs are targets for neonicotinoids. Imidacloprid acts as a partial agonist on cultured native Kenyon cells [17] and antennal lobe neurons [16], [48], [49]. Palmer et al. [18] showed that applications of imidacloprid or clothianidin depolarize Kenyon cells in isolated honeybee brains via nAChR activation with different efficacies. Similarly to *Drosophila* neurons, clothianidin has a higher potency for receptor activation than imidacloprid [15], [18]. However, both substances block the transmitter binding and thus act as blockers of cholinergic receptors upon prolonged applications in honeybees [17], [18,]. The nAChRs located in the mushroom body neurons are particularly relevant in our context, since the mushroom body in honeybees is a key structure in multimodal integration, learning and memory formation as well as memory retrieval [50]. Neonicotinoids interfere with cholinergic synaptic transmission in a complex way and may, thus, impair cognitive functions in the honeybee.

### Conclusion

Application of the three neonicotinoids imidacloprid, clothianidin and thiacloprid at sublethal doses interfered with navigation of honeybees, although it did not affect flight performance *per se* or the bees' motivation to return to the hive. The active and recently acquired navigation memory which would have brought the animals back to the hive (vector memory) is less compromised and appears even more stereotypical than in control bees because control bees tend to correct the displacement already during the vector flight. Thiacloprid treatment slowed the flight speed of bees while the other neonicotinoids did not affect flight speed. The second phase (homing) is impaired in treated bees reducing the probability of arriving at the hive, performing the correct turn at a salient landscape structure, and following a straight flight towards the hive. Since the homing phase in catch-and-release experiments documents the ability of the animal to activate a remote memory acquired during the exploratory orientation flights of a young bee and possibly during foraging flights before training to the feeder, we conclude that sublethal doses of the three neonicotinoids tested either block the retrieval of a remote memory or alter this form of navigation memory. These findings reinforce existing reservations about the application of neonicotinoids in plant protection [9], [30], [51], and uncover rather selective and highly relevant impairment of the foraging behavior of bees.

## Supporting Information

Archive S1 **This file archive contains 10 flight traces of control bees.**
(ZIP)

Archive S2 **This file archive contains 10 flight traces of clothianidin (0.2 µM) treated bees.**
(ZIP)

Archive S3 **This file archive contains 10 flight traces of imidacloprid (0.6 µM) treated bees.**
(ZIP)

Archive S4 **This file archive contains 5 flight traces of imidacloprid (0.9 µM) treated bees.**
(ZIP)

Archive S5 **This file archive contains 8 flight traces of thiacloprid (0.1 mM) treated bees.**
(ZIP)
