# Supplementary figures and images for: Neonicotinoids Interfere with Specific Components of Navigation in Honeybees
Source: PLoS One. 2014 Mar 19;9(3):e91364. doi: 10.1371/journal.pone.0091364 (PMC3960126; doi:10.1371/journal.pone.0091364)

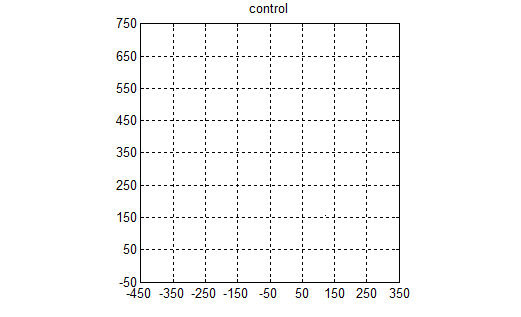

Supplement: Archive S1 — This file archive contains 10 flight traces of control bees. (ZIP) [file pone.0091364.s001.zip › control_S1/figureS01.gif]

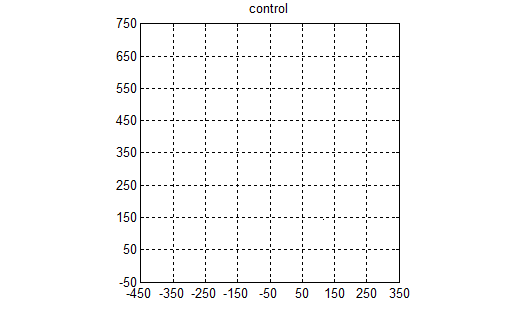

Supplement: Archive S1 — This file archive contains 10 flight traces of control bees. (ZIP) [file pone.0091364.s001.zip › control_S1/figureS02.gif]

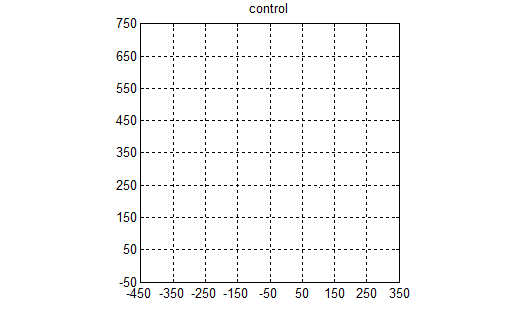

Supplement: Archive S1 — This file archive contains 10 flight traces of control bees. (ZIP) [file pone.0091364.s001.zip › control_S1/figureS03.gif]

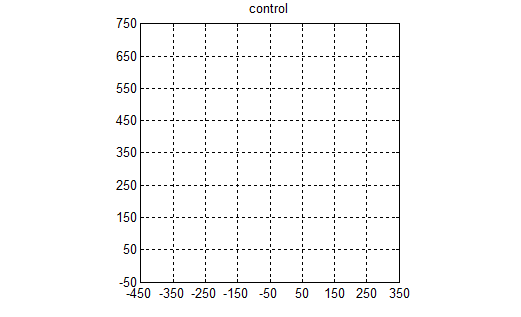

Supplement: Archive S1 — This file archive contains 10 flight traces of control bees. (ZIP) [file pone.0091364.s001.zip › control_S1/figureS04.gif]

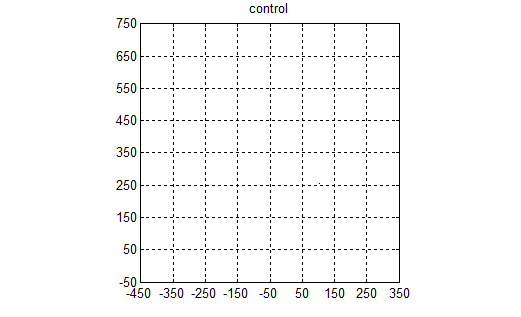

Supplement: Archive S1 — This file archive contains 10 flight traces of control bees. (ZIP) [file pone.0091364.s001.zip › control_S1/figureS05.gif]

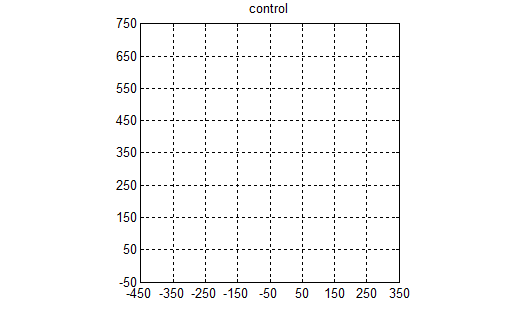

Supplement: Archive S1 — This file archive contains 10 flight traces of control bees. (ZIP) [file pone.0091364.s001.zip › control_S1/figureS06.gif]

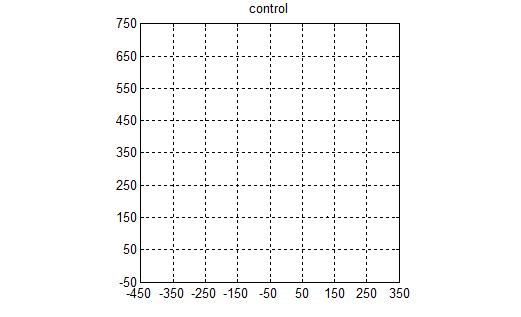

Supplement: Archive S1 — This file archive contains 10 flight traces of control bees. (ZIP) [file pone.0091364.s001.zip › control_S1/figureS07.gif]

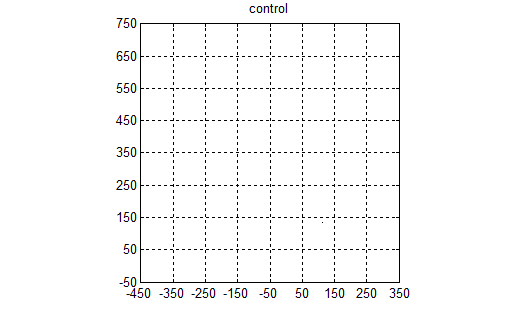

Supplement: Archive S1 — This file archive contains 10 flight traces of control bees. (ZIP) [file pone.0091364.s001.zip › control_S1/figureS08.gif]

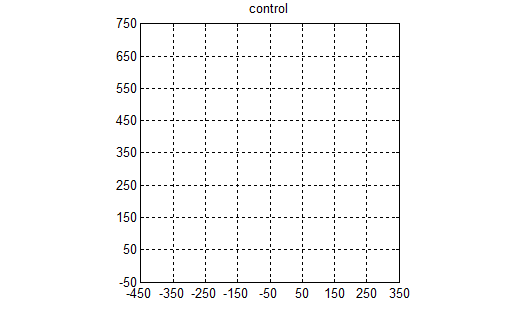

Supplement: Archive S1 — This file archive contains 10 flight traces of control bees. (ZIP) [file pone.0091364.s001.zip › control_S1/figureS09.gif]

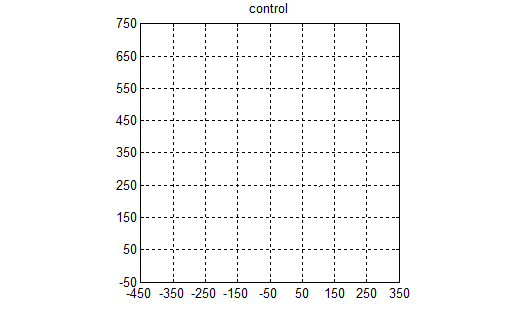

Supplement: Archive S1 — This file archive contains 10 flight traces of control bees. (ZIP) [file pone.0091364.s001.zip › control_S1/figureS10.gif]

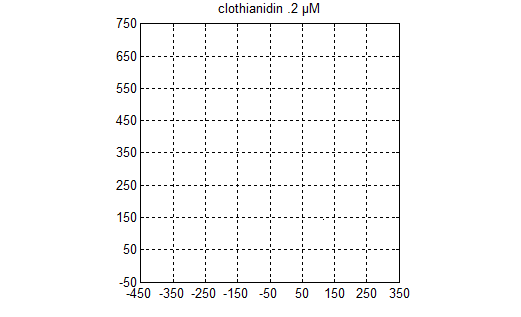

Supplement: Archive S2 — This file archive contains 10 flight traces of clothianidin (0.2 µM) treated bees. (ZIP) [file pone.0091364.s002.zip › clothianidin_S2/figureS11.gif]

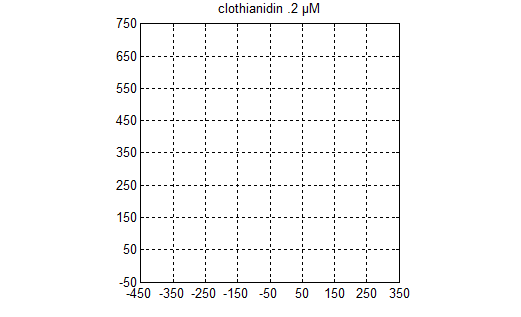

Supplement: Archive S2 — This file archive contains 10 flight traces of clothianidin (0.2 µM) treated bees. (ZIP) [file pone.0091364.s002.zip › clothianidin_S2/figureS12.gif]

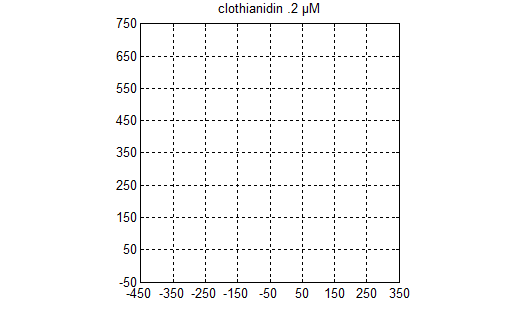

Supplement: Archive S2 — This file archive contains 10 flight traces of clothianidin (0.2 µM) treated bees. (ZIP) [file pone.0091364.s002.zip › clothianidin_S2/figureS13.gif]

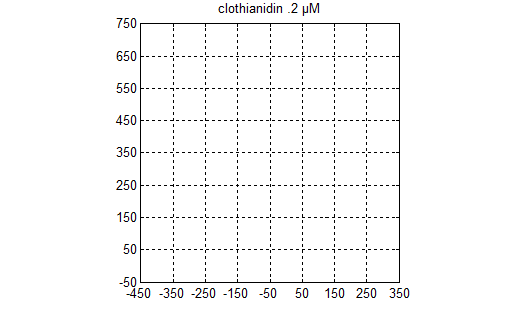

Supplement: Archive S2 — This file archive contains 10 flight traces of clothianidin (0.2 µM) treated bees. (ZIP) [file pone.0091364.s002.zip › clothianidin_S2/figureS14.gif]

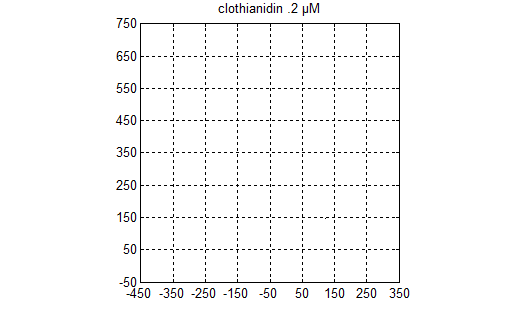

Supplement: Archive S2 — This file archive contains 10 flight traces of clothianidin (0.2 µM) treated bees. (ZIP) [file pone.0091364.s002.zip › clothianidin_S2/figureS15.gif]

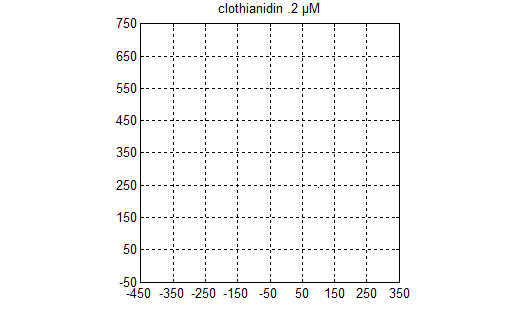

Supplement: Archive S2 — This file archive contains 10 flight traces of clothianidin (0.2 µM) treated bees. (ZIP) [file pone.0091364.s002.zip › clothianidin_S2/figureS16.gif]

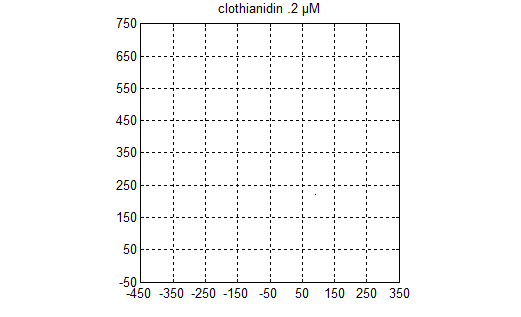

Supplement: Archive S2 — This file archive contains 10 flight traces of clothianidin (0.2 µM) treated bees. (ZIP) [file pone.0091364.s002.zip › clothianidin_S2/figureS17.gif]

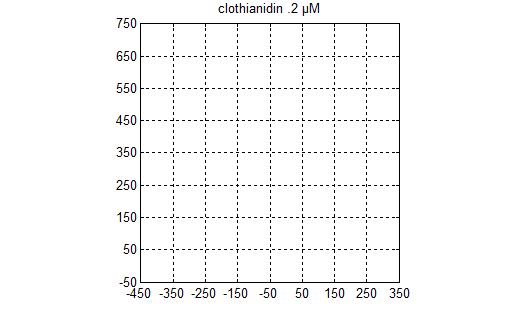

Supplement: Archive S2 — This file archive contains 10 flight traces of clothianidin (0.2 µM) treated bees. (ZIP) [file pone.0091364.s002.zip › clothianidin_S2/figureS18.gif]

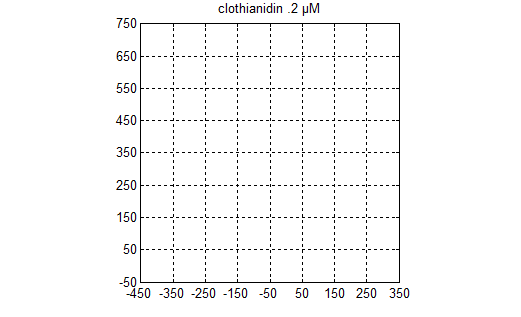

Supplement: Archive S2 — This file archive contains 10 flight traces of clothianidin (0.2 µM) treated bees. (ZIP) [file pone.0091364.s002.zip › clothianidin_S2/figureS19.gif]

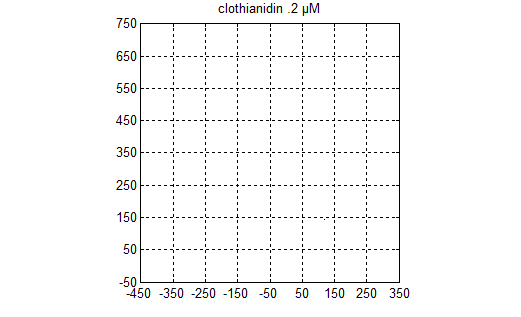

Supplement: Archive S2 — This file archive contains 10 flight traces of clothianidin (0.2 µM) treated bees. (ZIP) [file pone.0091364.s002.zip › clothianidin_S2/figureS20.gif]

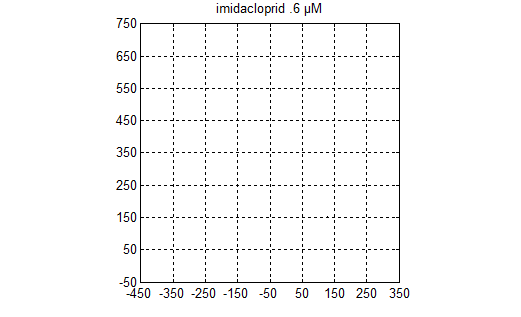

Supplement: Archive S3 — This file archive contains 10 flight traces of imidacloprid (0.6 µM) treated bees. (ZIP) [file pone.0091364.s003.zip › imidacloprid06_S3/figureS21.gif]

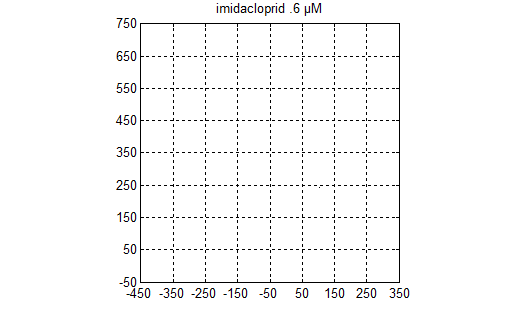

Supplement: Archive S3 — This file archive contains 10 flight traces of imidacloprid (0.6 µM) treated bees. (ZIP) [file pone.0091364.s003.zip › imidacloprid06_S3/figureS22.gif]

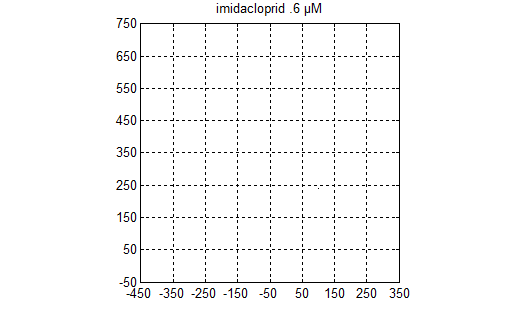

Supplement: Archive S3 — This file archive contains 10 flight traces of imidacloprid (0.6 µM) treated bees. (ZIP) [file pone.0091364.s003.zip › imidacloprid06_S3/figureS23.gif]

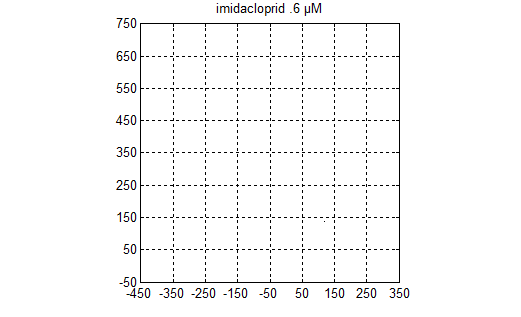

Supplement: Archive S3 — This file archive contains 10 flight traces of imidacloprid (0.6 µM) treated bees. (ZIP) [file pone.0091364.s003.zip › imidacloprid06_S3/figureS24.gif]

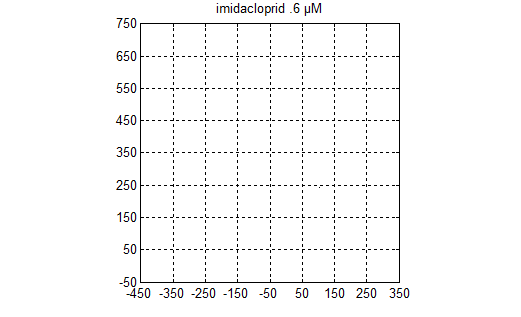

Supplement: Archive S3 — This file archive contains 10 flight traces of imidacloprid (0.6 µM) treated bees. (ZIP) [file pone.0091364.s003.zip › imidacloprid06_S3/figureS25.gif]

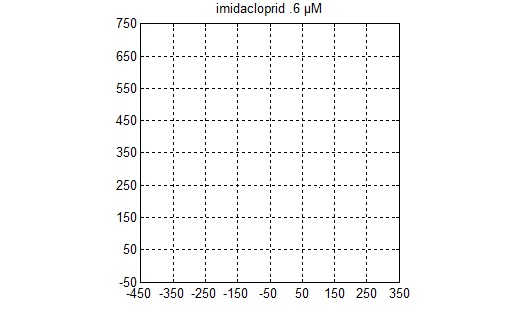

Supplement: Archive S3 — This file archive contains 10 flight traces of imidacloprid (0.6 µM) treated bees. (ZIP) [file pone.0091364.s003.zip › imidacloprid06_S3/figureS26.gif]

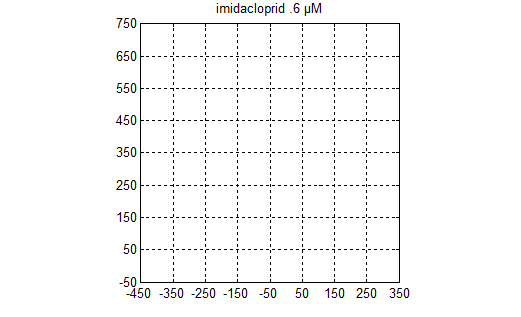

Supplement: Archive S3 — This file archive contains 10 flight traces of imidacloprid (0.6 µM) treated bees. (ZIP) [file pone.0091364.s003.zip › imidacloprid06_S3/figureS27.gif]

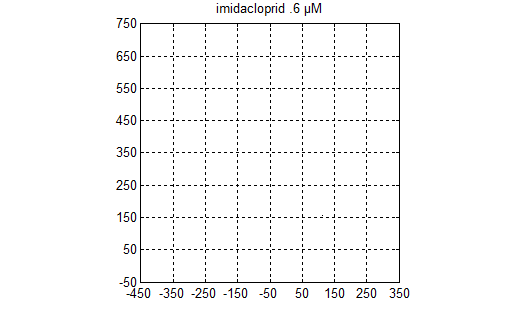

Supplement: Archive S3 — This file archive contains 10 flight traces of imidacloprid (0.6 µM) treated bees. (ZIP) [file pone.0091364.s003.zip › imidacloprid06_S3/figureS28.gif]

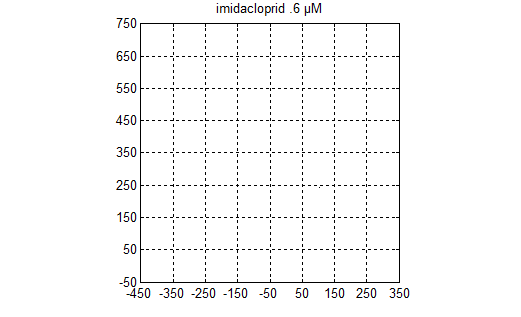

Supplement: Archive S3 — This file archive contains 10 flight traces of imidacloprid (0.6 µM) treated bees. (ZIP) [file pone.0091364.s003.zip › imidacloprid06_S3/figureS29.gif]

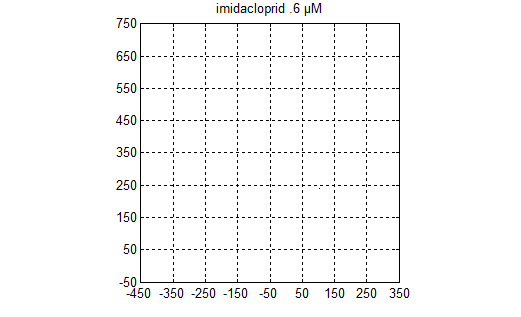

Supplement: Archive S3 — This file archive contains 10 flight traces of imidacloprid (0.6 µM) treated bees. (ZIP) [file pone.0091364.s003.zip › imidacloprid06_S3/figureS30.gif]

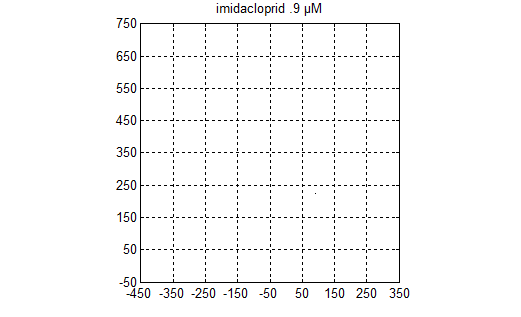

Supplement: Archive S4 — This file archive contains 5 flight traces of imidacloprid (0.9 µM) treated bees. (ZIP) [file pone.0091364.s004.zip › imidacloprid09_S4/figureS31.gif]

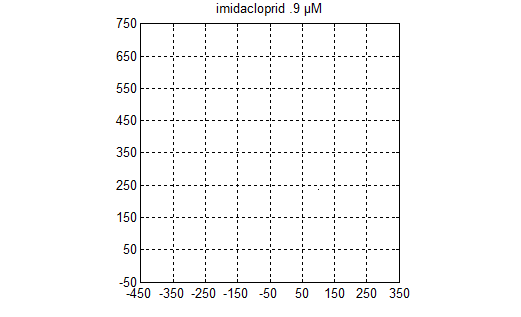

Supplement: Archive S4 — This file archive contains 5 flight traces of imidacloprid (0.9 µM) treated bees. (ZIP) [file pone.0091364.s004.zip › imidacloprid09_S4/figureS32.gif]

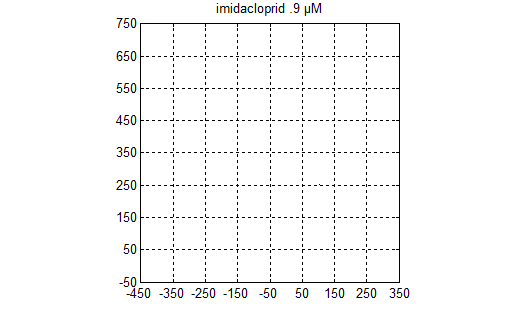

Supplement: Archive S4 — This file archive contains 5 flight traces of imidacloprid (0.9 µM) treated bees. (ZIP) [file pone.0091364.s004.zip › imidacloprid09_S4/figureS33.gif]

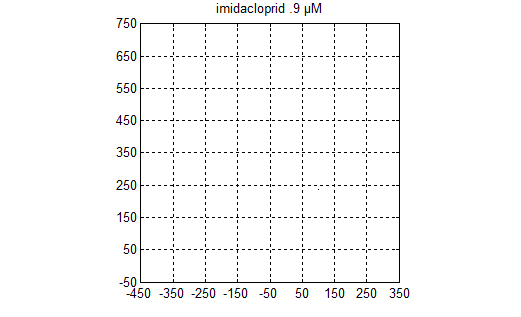

Supplement: Archive S4 — This file archive contains 5 flight traces of imidacloprid (0.9 µM) treated bees. (ZIP) [file pone.0091364.s004.zip › imidacloprid09_S4/figureS34.gif]

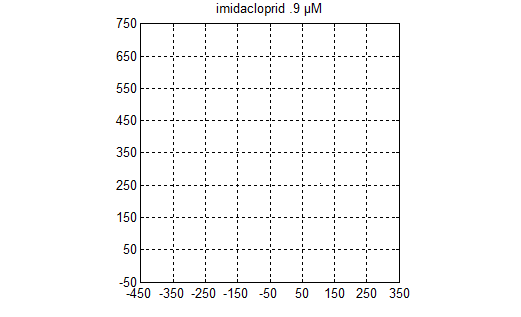

Supplement: Archive S4 — This file archive contains 5 flight traces of imidacloprid (0.9 µM) treated bees. (ZIP) [file pone.0091364.s004.zip › imidacloprid09_S4/figureS35.gif]

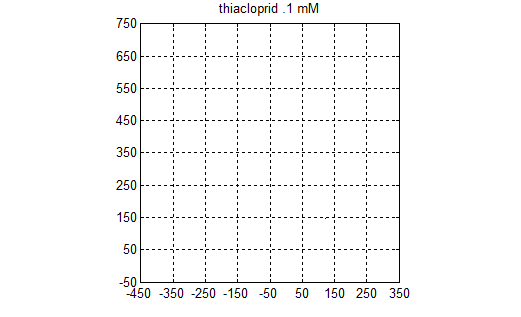

Supplement: Archive S5 — This file archive contains 8 flight traces of thiacloprid (0.1 mM) treated bees. (ZIP) [file pone.0091364.s005.zip › thiacloprid_S5/figureS36.gif]

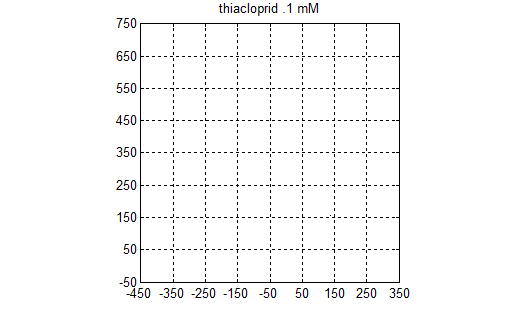

Supplement: Archive S5 — This file archive contains 8 flight traces of thiacloprid (0.1 mM) treated bees. (ZIP) [file pone.0091364.s005.zip › thiacloprid_S5/figureS37.gif]

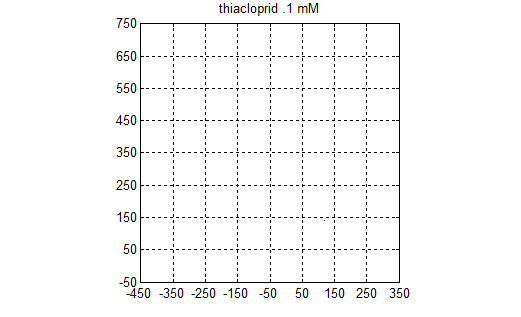

Supplement: Archive S5 — This file archive contains 8 flight traces of thiacloprid (0.1 mM) treated bees. (ZIP) [file pone.0091364.s005.zip › thiacloprid_S5/figureS38.gif]

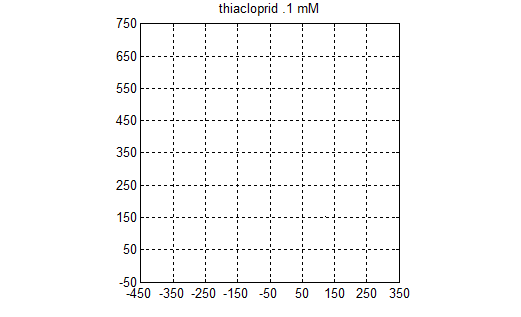

Supplement: Archive S5 — This file archive contains 8 flight traces of thiacloprid (0.1 mM) treated bees. (ZIP) [file pone.0091364.s005.zip › thiacloprid_S5/figureS39.gif]

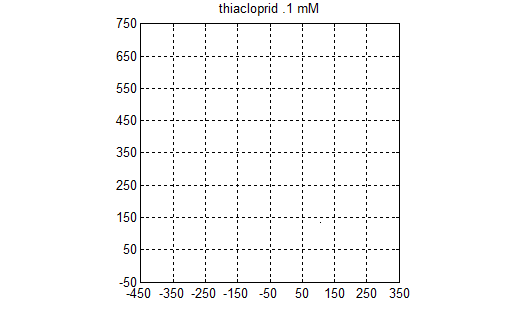

Supplement: Archive S5 — This file archive contains 8 flight traces of thiacloprid (0.1 mM) treated bees. (ZIP) [file pone.0091364.s005.zip › thiacloprid_S5/figureS40.gif]

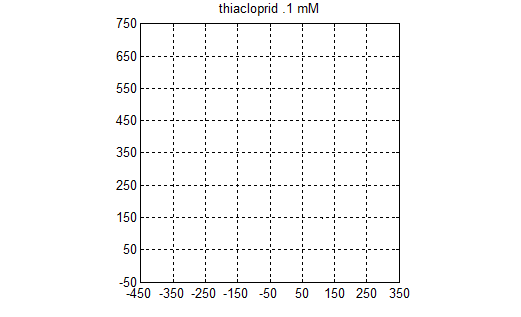

Supplement: Archive S5 — This file archive contains 8 flight traces of thiacloprid (0.1 mM) treated bees. (ZIP) [file pone.0091364.s005.zip › thiacloprid_S5/figureS41.gif]

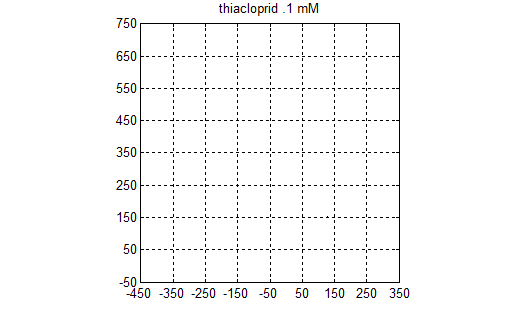

Supplement: Archive S5 — This file archive contains 8 flight traces of thiacloprid (0.1 mM) treated bees. (ZIP) [file pone.0091364.s005.zip › thiacloprid_S5/figureS42.gif]

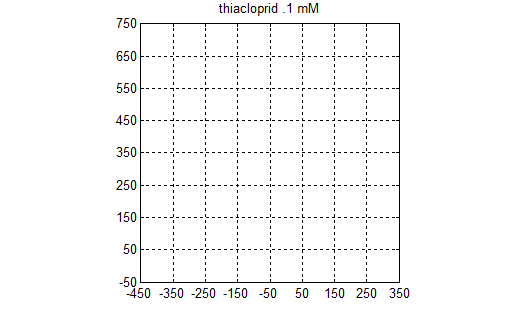

Supplement: Archive S5 — This file archive contains 8 flight traces of thiacloprid (0.1 mM) treated bees. (ZIP) [file pone.0091364.s005.zip › thiacloprid_S5/figureS43.gif]
